# Supplementary material for: Plasma Metabolite Response to Simple, Refined and Unrefined Carbohydrate-Enriched Diets in Older Adults—Randomized Controlled Crossover Trial
Source: Metabolites. 2022 Jun 15;12(6):547. doi: 10.3390/metabo12060547 (PMC9229237; doi:10.3390/metabo12060547)
Supplement: Supplementary file 1 [file metabolites-12-00547-s001.zip › metabolites-1715745-supplementary.pdf]

Plasma metabolite response to simple, refined and unrefined carbohydrate-enriched diets in older adults – randomized controlled crossover trial

Neil K. Huang,[1] Nirupa R. Matthan,[1] Gregory Matuszek,[2] Alice H. Lichtenstein[*, 1]

[1]Cardiovascular Nutrition Laboratory, Jean Mayer USDA Human Nutrition Research Center on Aging at Tufts University, Boston, MA 02111 U.S.A.

[2]Informatics Core Unit, Jean Mayer USDA Human Nutrition Research Center on Aging at Tufts University, Boston, MA 02111 U.S.A.

[*]Corresponding author: Alice H. Lichtenstein, D.Sc.,

[*]Correspondence:

Jean Mayer USDA Human Nutrition Research Center on Aging

Tufts University

711 Washington Street

Boston, MA 02111

Phone: (617)556-3127

Short title: Plasma biomarkers for dietary carbohydrate type

Journal Subject Terms: Dietary Composition, Dietary Biomarkers, Metabolomics, and Prevention/ Cardiovascular Disease

Supplementary Table S1. Composition of the 3 experimental diets.

Variables	Simple carb diet	Refined carb diet	Unrefined carb diet
		% energy	
Carbohydrate	63.9	60.8	61.7
Protein	13.4	14.2	14.7
Total fat	22.7	24.7	23.6
SFA	6.8	7.9	6.6
MUFA	8.7	9.4	9.2
PUFA	6.9	6.9	7.4
Trans	0.3	0.5	0.4
Cholesterol (mg/1000kcal)	108	99	99
Fiber (g/1000 kcal)	8.6	9.6	19.5
Soluble	2.2	2.8	4.4
Insoluble	6.4	6.8	15.1
Total sugar (g/1000kcal)	86.0	62.7	57.9
Starch (g/1000kcal)	44.6	47.1	50.9

The table was adapted from the parent study.[12] The data of carbohydrate, protein, and fat are presented as percentage of total calories, and other compositions are presented as indicated. MUFA, monounsaturated fatty acids; PUFA, polyunsaturated fatty acids; SFA, saturated fatty acids; carb, carbohydrate.

Supplementary Table S2. Cardiometabolic risk factors of the study participants at the end of each dietary phase.

Variables	Simple Carb	Refined Carb	Unrefined Carb
Weight, kg	85 ± 12	85 ± 12	86 ± 13
Body Mass Index, kg/m2	29.6 ± 2.9	29.7 ± 2.9	30.0 ± 3.0
Fasting glucose, mmol/L	5.3 (5.0-5.6)	5.2 (5.0-5.5)	5.1 (4.8-5.4)
Total cholesterol, mmol/L	5.2 (3.8-7.2)[b]	5.5 (4.0-7.6)[a]	5.2 (3.8-7.1)[b]
Non-HDL-C, mmol/L	4.0 (2.8-5.7)[b]	4.3 (3.0-6.1)[a]	4.0 (2.8-5.6)[b]
LDL-C, mmol/L	3.2 (2.2-4.5)[b]	3.4 (2.4-4.8)[a]	3.1 (2.2-4.4)[b]
VLDL-C, mmol/L	0.8 (0.5-1.3)	0.8 (0.5-1.4)	0.8 (0.5-1.3)
HDL-C, mmol/L	1.2 (1.0-1.5)	1.2 (1.0-1.5)	1.2 (1.0-1.5)
Triacylglycerol, mmol/L	1.7 (1.0-2.9)	1.9 (1.1-3.1)	1.7 (1.0-2.9)

Values were presented as mean±SD for weight and body mass index, and other continuous variables were presented as mean (95% confidence interval) (N=10). The table was adapted from the parent study [12]. Dietary effect in the parent study was assessed using repeated measure one-way ANOVA, followed by Tukey-Kramer post hoc analysis, $p<0.01$, a>b. HDL-C, high-density lipoprotein-cholesterol; LDL-C, low-density lipoprotein-cholesterol; TC/HDL-C, the ratio of total cholesterol: high-density lipoprotein-cholesterol; VLDL-C, very low-density lipoprotein-cholesterol.

Supplementary Table S3. The electrospray ionization mode, m/z value and retention time for the metabolites with Variable importance projection scores > 1.7.

Metabolites	Category	VIP score	ESI mode	m/z; Quant m/z*	RT; RT index*
Phenylethylamine	Amino acid	3.03	-	174*	513300*
Cysteine	Amino acid	3.00	-	220*	500185*
Betaine	Xenobiotics	2.84	ESI (+)	118.0861	6.92
Pipecolic acid	Amino acid	2.83	ESI (+)	130.084	7.46
TMAO	Amino acids	2.57	ESI (+)	76.0756	5.29
3-Methylhistidine	Amino acids	2.49	ESI (-)	168.0773	9.16
PC 38:3	Phospholipids	2.47	ESI (+)	812.6147	5.77
TG 42:0	Lipids	2.45	ESI (+)	740.6776	9.43
TG 51:1(TG 16:0_17:0_18:1)	Lipids	2.45	ESI (+)	864.8098	11.0
Conduritol-beta-epoxide	Xenobiotics	2.37	-	318*	675635*
N-acetylglycine	Amino acids	2.37	ESI (-)	116.0359	7.19
TG 45:1(TG 12:0_16:0_17:1)	Lipids	2.29	ESI (+)	780.7114	9.70
PI 36:4	Phospholipids	2.25	ESI (-)	857.5192	4.34
TG 46:2	Lipids	2.24	ESI (+)	792.7114	9.52
TG 44:0	Lipids	2.23	ESI (+)	768.7067	9.92
Pipecolinic acid	Amino acids	2.23	-	156*	404121*
Coniferyl aldehyde	Xenobiotics	2.17	ESI (-)	177.0556	1.32
TG 54:5(TG 18:1_18:2_18:2)	Lipids	2.15	ESI (+)	898.7851	10.1
3-hydroxybutyric acid	Ketone	2.12	ESI (-)	103.0412	1.87
LPC 20:3	Phospholipids	2.11	ESI (+)	546.3558	1.37
3,5-Dihydroxyphenylglycine	Amino acids	2.10	ESI (+)	184.0694	7.99
TG 47:1|TG 14:0_15:0_18:1	Lipids	2.06	ESI (+)	808.7356	10.2
TG 49:0	Lipids	2.05	ESI (+)	838.7961	11.0
Lyxitol	Carboxylic acids	2.05	-	217*	573587
10-Hydroxydecanoic acid	Lipids	2.05	ESI (-)	187.1341	1.27
Lactic acid	Carboxylic acids	2.00	-	191*	217657*
2-Methylglutaric acid	Amino acids	1.99	ESI (-)	145.0509	2.18
3-Hydroxybutyrylcarnitine	Lipids	1.98	ESI (+)	248.1492	7.26
Tetradecylsulfate	Xenobiotics	1.97	ESI (-)	293.1784	1.10
TG 49:1|TG 15:0_16:0_18:1	Lipids	1.97	ESI (+)	836.788	10.6
TG 46:0	Lipids	1.96	ESI (+)	796.7418	10.4
TG 53:1|TG 17:0_18:0_18:1	Lipids	1.95	ESI (+)	892.843	11.4
Quinolone	Xenobiotics	1.95	ESI (+)	146.0634	1.31
TG 54:3|TG 18:0_18:1_18:2	Lipids	1.94	ESI (+)	902.8325	10.9
TG 47:2|TG 14:0_15:0_18:2	Lipids	1.92	ESI (+)	806.7283	9.75
TG 54:8|TG 18:2_18:3_18:3	Lipids	1.91	ESI (+)	892.7405	8.97
PC 38:2	Phospholipids	1.90	ESI (+)	814.6323	6.18
TG 47:0|TG 15:0_16:0_16:0	Lipids	1.89	ESI (+)	810.754	10.6
Trigonelline	Vitamins	1.89	ESI (+)	138.0544	5.72
Indole-3-propionic acid	Xenobiotics	1.88	-	202*	732249*

TG 46:3 Isomer A	Lipids	1.88	ESI (+)	790.693	9.04
PC 33:1	Phospholipids	1.87	ESI (+)	746.5717	5.14
Deoxypentitol	Carbohydrate	1.86	-	231*	528774*
TG 51:0|TG 16:0_17:0_18:0	Lipids	1.85	ESI (+)	866.8299	11.36
TG 48:1	Lipids	1.85	ESI (+)	822.763	10.4
Riluzole	Xenobiotics	1.81	ESI (-)	232.9955	1.04
Proline	Amino acids	1.81	-	114.0566	7.52
Nateglinide	Xenobiotics	1.81	ESI (-)	316.188	1.18
PC 30:0	Phospholipids	1.81	ESI (+)	706.5359	4.77
PC 36:1	Phospholipids	1.81	ESI (+)	788.615	6.09
Indoxyl sulfate	Amino acids	1.80	ESI (-)	212.0029	1.38
TG 42:1	Lipids	1.79	ESI (+)	738.6621	8.93
Hexadecanedioic acid	Xenobiotics	1.78	ESI (-)	285.2066	1.24
TG 52:5	Lipids	1.78	ESI (+)	870.7491	9.79
Glutamine	Amino acids	1.77	ESI (+)	147.0772	8.29
Mannitol	Carbohydrate	1.76	ESI (-)	181.0709	7.54
1,2-Diamino-2-methylpropane	Xenobiotics	1.76	ESI (+)	72.0806	7.38
3-Hydroxybutyric acid	Ketone	1.75	ESI (-)	103.0412	1.87
Homovanillic acid	Hormone	1.75	ESI (-)	181.0504	1.41
PI 40:6	Phospholipids	1.74	ESI (-)	909.5487	4.75

Asterisk indicates the data were from gas chromatography time-of-flight mass spectrometry. ESI, electrospray ionization; PC, phosphatidylcholine; PI, phosphatidylinositol; RT, retention time; TG, triacylglycerol.

Supplementary Table S4. Pearson Correlation Coefficient between top 5 plasma metabolites and cardiometabolic risk factors – simple, refined and unrefined carbohyrate diets.

Plasma metabolite	TC	TG	VLDL	LDL	HDL
	Simple carb diet				
Phenylethylamine	-0.201	-0.251	-0.251	-0.174	-0.040
Cysteine	0.342	0.590	0.593	0.248	0.042
Betaine	-0.282	-0.384	-0.369	-0.289	0.072
Pipecolic acid	0.599	0.247	0.262	0.509	0.600
3-Methylhistidine	0.291	0.255	0.262	0.314	-0.001
	Refined carb diet				
Phenylethylamine	-0.295	-0.282	-0.267	-0.223	-0.106
Cysteine	-0.311	-0.325	-0.314	-0.298	0.008
Betaine	0.007	0.399	0.388	-0.107	-0.231
Pipecolic acid	-0.371	-0.084	-0.077	-0.388	-0.167
3-Methylhistidine	-0.127	-0.145	-0.160	-0.101	-0.024
	Unrefined carb diet				
Phenylethylamine	-0.143	0.006	-0.008	-0.053	-0.330
Cysteine	-0.291	0.086	0.067	-0.273	-0.307
Betaine	-0.086	0.009	0.012	-0.083	-0.055
Pipecolic acid	0.095	-0.182	-0.178	0.083	0.246
3-Methylhistidine	0.021	0.212	0.206	-0.032	-0.021

Values were presented as correlation coefficient (*r*). No significant correlation was found. HDL, high-density lipoprotein; LDL, low-density lipoprotein; TC, total cholesterol; TC, triglyceride; carb, carbohydrate.

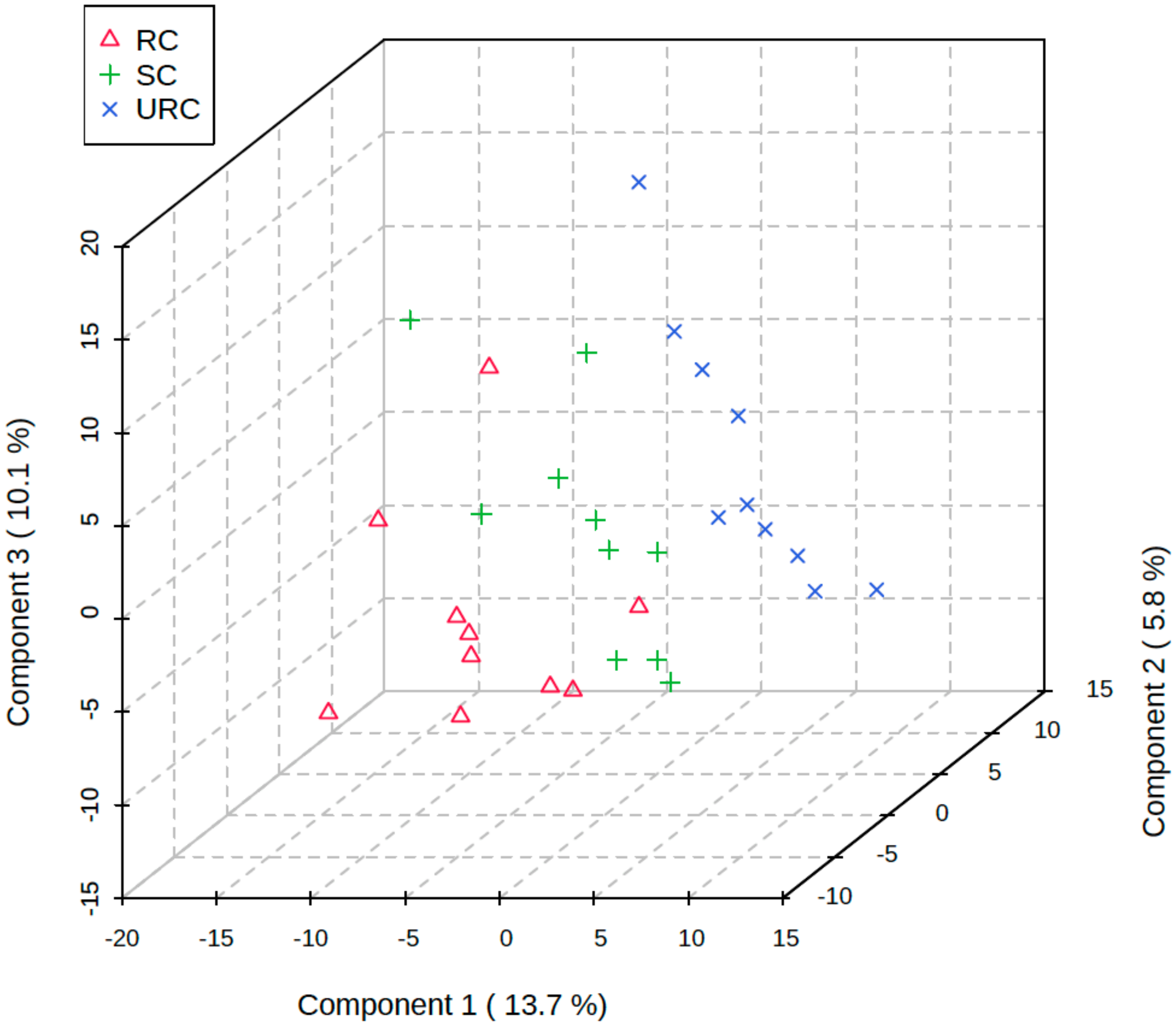

Supplementary Figure S1. Partial least squares discriminant analysis (PLS-DA) of the plasma metabolites in participants after they received the simple carbohydrate, refined carbohydrate and unrefined carbohydrate diets.

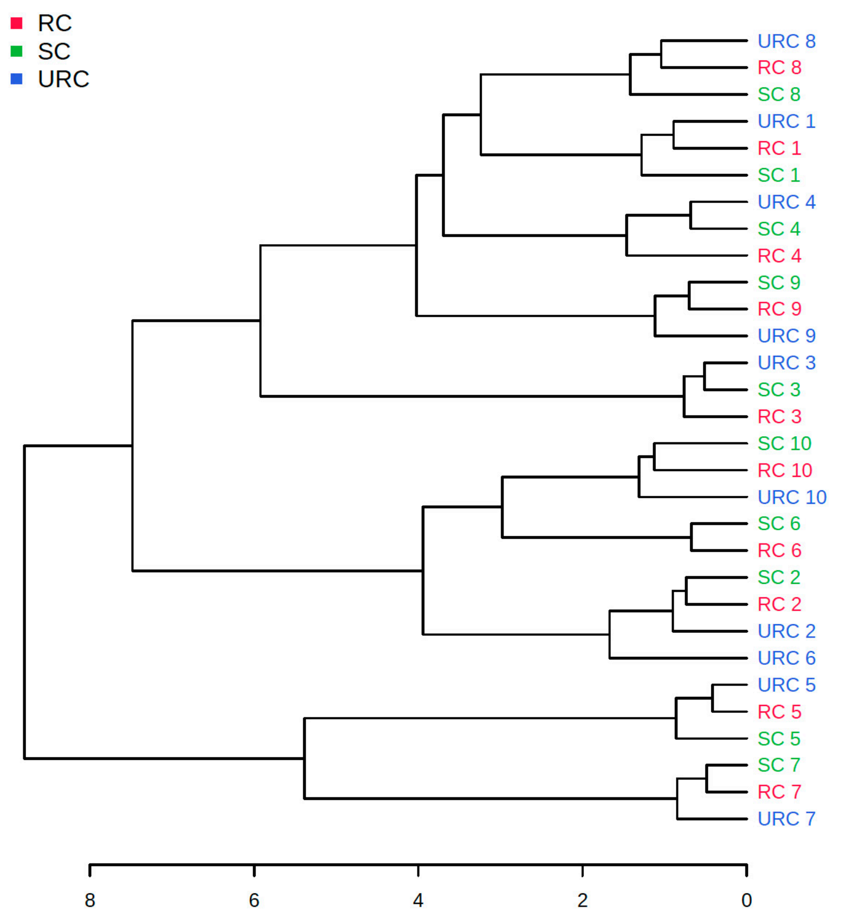

Supplementary Figure S2. Hierarchical clustering of the plasma metabolites in participants after they received the simple carbohydrate, refined carbohydrate, and unrefined carbohydrate diets.
